# The existence and characteristics of rats and shrews in endemic leptospirosis areas and types of ectoparasites: a case study of West Jakarta, Indonesia

**DOI:** 10.12688/f1000research.47068.1

**Published:** 2021-04-30

**Authors:** Dewi Susanna, Rusyda Ihwani Tantia Nova, Laura Rozek

**Affiliations:** 1 Environmental Health, Universitas Indonesia, Depok, Indonesia; 2 Environmental Health Sciences, Global Public Health, and Nutrition, University of Michigan School of Public Health, Michigan, USA

**Keywords:** species, rats, ectoparasite, shrew

## Abstract

**Background: **This study aimed to determine the presence and species of the rats and shrews that can potentially cause leptospirosis in West Jakarta, Indonesia, and the species of ectoparasites found in them.

**Methods:** The research was a descriptive study employing a cross-sectional approach. The study population was all species of rats and shrews in the region and the sample collection technique used was purposive sampling. The traps were installed in the homes of respondents who had suffered from leptospirosis and their closest neighbors, with a total of 521 traps. Leptospirosis data based on secondary data was obtained from West Jakarta Health Office (2016-August 2019). The technique for catching rats involved using humane live traps, while the identification of the rats and ectoparasites was done in the laboratory.

**Results:** It was found that more rats were caught in Cengkareng Timur sub-district, Cengkareng District, with a percentage of 14.8%, while the least in Duri Kepa, Kapuk, Kedaung Kali Angke and Kedoya Utara with a percentage of 3.7%. The rats were mostly found in East Cengkareng Sub-District, with the most common type being *Rattus rattus* (74.1 %) and the least *Suncus murinus* (11.1%); more male rats were caught (66.7%) than female (33.3%). The type of ectoparasite found in the rats was fleas. *Xenopsylla cheopis* was the most common type, at 83.3% and more fleas were male, at 66.7%. The most common rat species was *Rattus rattus*. The ectoparasite most commonly found in them was the female flea *Xenopsylla cheopis.*

**Conclusions:** *Rattus rattus* and *Xenopsylla cheopis* were found in an East Cengkareng sub-district. Surveys, monitoring, and control of rats and ectoparasites are essential for the preparedness and development of an early warning system of possible diseases that they can cause.

## Introduction

Rodents such as rats and shrews can carry various bacteria and viruses that can cause infections in humans. They are thought to be a reservoir of 30% of zoonotic pathogens, including several viruses, bacteria, and parasites. Rats are one of the most important components of an ecosystem. Their presence is very widespread, representing 40% of all mammal species ( Churakov *et al.*, 2010). They can be useful as food for some mammals and predatory birds ( Tobin & Fall, n.d.), but their presence in the ecosystem can cause various losses in sectors such as agriculture and health ( Tobin & Fall, n.d.). Rats are a reservoir of various diseases, and as animals that cannot be separated from human life, they can cause various health problems in humans and pets and other wildlife ( Tobin & Fall, n.d.). They are also an important host for ectoparasites and have a close relationship, such as lice, fleas, and mites ( Kiffner, Vor, Hagedorn, Niedrig, & Rühe, 2011). They can produce a variety of associations influenced by the host and parasite species and the biotic and abiotic environment ( Buchholz & Dick, 2017). The rate of rat ectoparasite infestation can reach 66.6%, and ectoparasites are vectors for various diseases which can cause health problems ( Zendehfili, Zahirnia, Maghsood, Khanjani, & Fallah, 2015).

Leptospirosis can cause death, with the case fatality rate in humans of 5-30% ( CDC, 2018). It is a disease caused by Leptospira bacteria ( World Health Organization, 2003). *Leptospira interrogans* and *Leptospira borgpetersenni* are present in many populations of rats and have been confirmed to cause leptospirosis in humans ( Cosson *et al.*, 2014). The disease can be transmitted through water or soil contaminated by the urine of rats infected with Leptospira bacteria and direct contact with infected animals ( Centers for Disease Control and Prevention, 2018; Haddis, 2004; World Health Organization, 2003). Leptospira bacteria can enter the human body through mucous membranes, wounds, or blisters on the skin ( Centers for Disease Control and Prevention, 2018; Haddis, 2004; World Health Organization, 2003). In several previous studies, it has been found that *Rattus norvegicus* is the rat species that is the main reservoir of Leptospira bacteria ( Marcos Tucunduva de Faria *et al.*, 2013; Pui, Bilung, Apun, & Su’ut, 2017).

Environmental conditions and their habitat greatly influence the presence of rats; each species has a different habitat. *Mus musculus* is a rat species that like to live in homes, outbuildings, and shops ( Global Invasive Species Database, 2015). *Rattus rattus* is spread in forests and can also live in and around buildings, both underground and above ground ( Csurhes, 2012). *Rattus norvegicus* is a species that is widespread in places such as sewers, agricultural and horticultural land, grasslands, and the interior part of the region ( Hausser & de Roguin, 1995). The rat population will continue to increase to the level of capacity accommodated by their habitat ( Jackson, 1972). Moreover, the rat population will also greatly depend on the availability of food and predators' presence in their habitat.

West Jakarta is one of the five administrative cities in the Special Capital Region of Jakarta, Indonesia; its center is in Kembangan. West Jakarta has an area of 129.54 km ^2^ and eight subdistricts, namely Taman Sari, Tambora, Kembangan, Kalideres, Cengkareng, Palmerah, Kebun Jeruk, and Grogol Petamburan Districts ( *Badan Pusat Statistik Administrasi Jakarta Bara*t, 2019). West Jakarta is one of the areas with 70 cases from January 2016 to August 2019 *(Suku Dinas Kesehatan Jakarta Barat,* 2019). The population density in West Jakarta in 2018 was 19,757 inhabitants/km ^2^, with an average population per household of four *(Badan Pusat Statistik Administrasi Jakarta Barat,* 2019). This means that West Jakarta is a densely populated area, one of the conditions that rats highly favor. This research aims to establish the presence and species of the rats and shrews that can potentially cause leptospirosis in West Jakarta and the species of ectoparasite found in them. The research could be used as the basis for an early alert system for various diseases that rats can carry, and also as a preliminary study to ascertain which rat species are the main reservoirs of leptospirosis in West Jakarta.

## Methods

### Study site

This research describes the presence and species of the rats and shrews in an endemic leptospirosis area in West Jakarta, employing a cross-sectional approach. The study population comprised all species of rats and shrews in the region. The research was conducted in December 2019 and consisted of all people diagnosed with leptospirosis, which referred to the doctor's diagnosis results through clinical reports and laboratory tests that were reported and recorded in the West's work area Jakarta Health Office from January 2016 to August 2019. The sample collection technique used was purposive sampling, involving the installation of rat traps at the homes of participants who had suffered from leptospirosis and their closest neighbors.

### Rat catching

The rodents were caught using a live trap. This live trap has no brand made from wire 34 cm length, 20 width, and 15 heights. Each house had two live traps installed over two consecutive days with a total of 128 houses (16 houses in Kembangan District, 10 houses in Grogol Petamburan District, 24 houses in Cengkareng District, 18 houses in Kebun Jeruk District, 30 houses in Kalideres District, 12 houses in Palmerah District, and 18 houses in Tambora District) and 512 traps (64 live traps in Kembangan District, 40 live traps in Grogol Petamburan District, 96 live traps in Cengkareng District, 72 live traps in Kebun Jeruk District, 120 live traps in Kalideres District, 48 live traps in Palmerah District, and 72 live traps in Tambora District). The bait used was salted fish, which was changed every day during the capture process. In each house, as many as two traps were installed in the place where small mammals are suspected of passing by, evidenced by signs of rodents such as footprints, rat droppings, the smell of rodents, bite marks, digs/earthen holes, and the sound of small mammals. The traps were installed in the afternoon between 15.00 and 17.00 WIB, and the rodents were collected in the morning between 07.00 and 09.00. The traps managed to ensnare rats on the first day were taken and replaced with new ones. Trapped rodents were then labeled by name, serial number, head of families (head of household), district, community neighborhood (RW), neighborhood unit (RT), date, and the day the rat was trapped. The rats caught on the first and second days were collected and stored in the respondent's home to be collected the next day by the researchers. The rodents were put into white sacks and their traps and then taken to the Tanjung Priok Class I Port Health Office (KKP) for identification.

### Rat identification

The rats were put into an airtight plastic bag and anesthetized using chloroform with a dose of 5 – 10 mL. The chloroform was poured into cotton, and the cotton was put into the plastic bag. After the chloroform was added, the rats were left for 10 to 15 minutes until the rats and shrews passed out or died. All rats died from the chloroform dose. Identifying the rats was performed by using external morphological signs such as body length, tail length, back foot length, ear length, head length, mammae, and body weight. Besides, the hair color, type, and size were also considered and then matched with the rat identification key ( Badan Penelitian dan Pengembangan Kesehatan & Kementerian Kesehatan Republik Indonesia, 2016). After the identification, the rats and shrews were put in plastic bags and buried with a depth of 2,5 m. Before being buried, the plastic bag was disinfected using alcohol 70%. It will handle by Tanjung Priok Class I Port Health Office or *Kantor Kesehatan Pelabuhan* (KKP) according to existing procedures ( Direktorat Jenderal Pencegahan dan Pengendalian Penyakit Tular Vektor dan Zoonotik, 2019).

### Ectoparasite identification

The rats that had fainted or died were placed in a white tray, then combed with a flea comb. Ectoparasites that fell into the tray were taken using tweezers, placed into a bottle of 70% alcohol, then labeled according to the area where they were caught. Ectoparasites with hard skin such as fleas were first soaked in 10% KOH solution for 24 hours. They were then put into six Petri dishes, previously filled with alcohol, aqua dest, and xylol solution (6 ^th^ Petri dish) alternately with a transfer time span of 2 minutes for each Petri dish. The fleas were then placed inside a glass object and covered with a glass deck. Each part of the edge of the glass deck was glued to close tightly to the glass object. The ectoparasites were then examined under a microscope and matched to the ectoparasite identification key ( Mathison & Pritt, 2014) in Tanjung Priok Class I Port Health Office, Jakarta.

### Ethical issues

All the procedures complied with the National Research Committee's ethical standards. The study was approved by the Research and Community Engagement Ethical Committee of the Faculty of Public Health, Universitas Indonesia No. 650/UN2.F10/PPM.00.02/2019. All efforts were made to ameliorate harm to the animals by ensuring the animal welfare by following the all procedures in accordance with the technical instructions for rats surveillance laboratory-based and the guidance for rats and mice control (Kementerian Kesehatan Republik Indonesia, 2015).

## Results

Based on the results of the study, it was found that more rats were caught in Cengkareng Timur sub-district, Cengkareng District, with a percentage of 14.8% of the total ( Table 1) ( Susanna, Nova & Rozek, 2021). Based on the result, it can be seen that the rat species most commonly found in the West Jakarta area was *Rattus rattus* (74.1%), *Rattus norvegicus* (14.8), *Suncus murinus* (11.1%), and more male rats were caught, equal to 66.7% (female 33.3%) ( Table 2). The only type of ectoparasite found in the rats in the West Jakarta area was the flea ( Table 3). *Xenopshilla cheopis* was the most common type (83.3%) and *Xenopsylla astia* (16.7%). More fleas were male (66.7%) than female (33.3%).

**Table 1.** Frequency distribution of rats and shrews caught in the West Jakarta area, November 2019.

Region	Number of rats caught	Percentage (%)
West Cengkareng sub-district	2	7.4
East Cengkareng sub-district ^*^	4	14.8
Duri Kepa sub-district	1	3.7
Kamal sub-district	3	11.1
Kapuk sub-district	1	3.7
Kedaung Kali Angke sub-district	1	3.7
Nort Kedoya sub-district	1	3.7
Nort Kembangan sub-district	3	11.1
South Meruya sub-district	2	7.4
Rawa Buaya sub-district	2	7.4
Semanan sub-district	2	7.4
South Tanjung Duren sub-district	2	7.4
Tegal Alur sub-district	3	11.1
Total	27	100.0

* The region was found more rats and Shrews.

**Table 2.** Characteristics of rats and shrews caught in West Jakarta, November 2019.

Species	Gender	Body length (mm)	Tail length (mm)	Rear feet length (mm)	Earlobe length (mm)	Head length (mm)	Mammae	Weight (Grams)
*Rattus rattus*	Male	70	110	10	10	30	0	31
*Rattus rattus*	Male	75	110	15	15	20	0	33
*Rattus rattus*	Male	180	185	25	20	40	0	324
*Rattus rattus*	Male	70	115	10	15	40	0	26
*Rattus rattus*	Female	130	155	20	15	50	6	85
*Rattus rattus*	Male	120	150	15	10	35	0	89
*Rattus rattus*	Male	130	140	15	15	30	0	89
*Rattus rattus*	Male	120	160	20	20	40	0	103
*Rattus rattus*	Female	130	180	15	20	30	8	121
*Rattus rattus*	Male	90	130	10	10	30	0	45
*Rattus rattus*	Male	90	150	10	10	30	0	46
*Rattus rattus*	Male	180	145	20	20	55	0	210
*Rattus rattus*	Male	130	140	15	20	35	0	81
*Rattus rattus*	Male	130	165	20	35	20	0	188
*Rattus rattus*	Female	130	180	20	30	20	10	145
*Rattus rattus*	Female	100	150	15	15	25	10	71
*Rattus rattus*	Male	110	170	15	15	25	0	68
*Rattus rattus*	Male	120	170	20	15	35	0	86
*Rattus rattus*	Female	130	160	15	15	40	12	122
*Rattus rattus*	Male	85	110	15	15	35	0	49
*Rattus norvegicus*	Female	170	165	25	20	50	14	287
*Rattus norvegicus*	Female	190	180	30	25	50	12	429
*Rattus norvegicus*	Male	160	200	35	20	55	0	245
*Rattus norvegicus*	Male	180	170	30	25	50	0	231
*Suncus murinus*	Male	70	65	10	50	30	0	38
*Suncus murinus*	Female	90	65	15	10	40	6	50
*Suncus murinus*	Female	70	55	10	5	30	0	25

**Table 3.** Frequency distribution of ectoparasites in rats caught in West Jakarta, November 2019.

Ectoparasite	Number	Percentage (%)
**Fleas**		
*Xenopsylla astia*	3	16.7
*Xenopsylla cheopis*	15	83.3
**Total**	**18**	**100.0**

## Discussion

Cengkareng district is the administrative area of West Jakarta with the highest population, comprising 514,416 people ( Badan Pusat Statistik Kota Jakarta Barat, 2019). It has 26.54 km ^2^ and includes six sub-Districts; Cengkareng Timur sub-District is one of the densely populated areas in Cengkareng district ( Badan Pusat Statistik Kota Jakarta Barat, 2019). Based on the research results, rats are most commonly found in the East Cengkareng sub-District. This study found different results to previous studies, which found that rats were more commonly found in agricultural areas (58.0%) ( Munõz-Zanzi, Mason, Encina, Gonzalez, & Berg, 2014). These different results could be caused by regional differences and the categories of the research areas. Previous studies [25] have researched three locations, namely agricultural areas, rural areas, and slums, while this study was only conducted in West Jakarta, without categorizing the area.

The presence of rats in an area depends on ecology, vegetation, food availability, and predators' presence. Also, the presence of rats also correlates with the number of tree species; their number will tend to increase in areas with high tree diversity ( Madden *et al.*, 2019). However, in this study, the level of tree diversity did not affect rats' presence, as the habitat of those caught was in homes and gutters. Moreover, Cengkareng Timur sub-district is not an area that has a diversity of trees. Seasons also have an important influence on rats' presence; house or commensal rats will be more common in the dry season ( Panti-May, Hernández-Betancourt, Ruíz-Piña, & Medina-Peralta, 2012). In this study, the rats were found in densely populated areas, and the process of catching them took place in the dry season so that more would be caught. In the dry season, the availability of rat food is higher, especially leftovers from processed household products, as well as from some home industries in the Eastern Cengkareng sub-district.

In this study, the rat species most commonly found was *Rattus rattus* (black rats). This species was commonly found because catching the rats and setting traps was commonly found in participants' homes. *Rattus rattus* is a species whose habitat is forests and homes ( Csurhes, 2012). The species can also be found in natural and semi-natural habitats ( The ICUN Red List, 2017). It is an arboreal animal that can climb. *Rattus rattus* favors lowlands that are less than 250 meters above sea level ( Isnaini, 2008). The environmental conditions greatly affect the presence of *Rattus rattus* in the ecosystem, for example, the availability of food sources. Food, organic waste that is not stored or properly disposed of, gardens that are not well managed; and the presence of pets and livestock can affect the number of rats, including the *Rattus rattus* species ( Feng & Himsworth, 2014).

The presence of rats in the community environment can cause various health problems, one of which is leptospirosis, a disease caused by Leptospira bacteria ( Centers for Disease Control and Prevention, 2018; Haake, David A, & Levett, 2015; World Health Organization, 2003). Rats are the most important reservoir of Leptospira bacteria. Previous studies have found a similarity between the PFGE pattern and gyrB sequence in Leptospira bacteria isolated from humans and rats in Luzon, Philippines ( Villanueva *et al.*, 2014). This indicates that rats infected with Leptospira bacteria can cause leptospirosis in humans ( Villanueva *et al.*, 2014). The prevalence rate of Leptospira bacteria in each rat species is different. Based on the results of previous studies, it is known that Leptospira spp positively infects up to 17.8% of the *Rattus rattus* species., 30.3% of the *Rattus norvegicus* species, 10.9% of the *Rattus exulans* species, 19.3% of the *Rattus argentivente*r species, and 3.4% of the *Rattus tanezumi* species ( Id, Shiokawa, & Id, 2019; Koizumi *et al.*, 2009). From these results, it is clear that *Rattus norvegicus* is the most infected species with the Leptospira bacteria, which causes leptospirosis in humans. However, to establish which species of rats are most responsible for carrying Leptospira bacteria and causing leptospirosis, further research is needed.

In addition to the presence of rats, ectoparasites can also cause other health problems for humans. One disease that can be caused by rat ectoparasites (fleas) is pes (plague) ( Illinois Department of Public Health, n.d.; Nurisa, 2005). Based on the study findings, out of the 27 rats caught, some had ectoparasites such as fleas. The most common type of flea was *Xenopsylla cheopis* (male). The results of this study are in line with those of previous study by Maulana *et al.*, which also found that several species of rats carried ectoparasites, with the most commonly found type being *Xenopsylla cheopis* (81.1%) ( D, 2012). In another study by Kia et al, it was also found that one of several fleas found in *Rattus rattus* and *Rattus norvegicus* was related to the transmission of plague. The most abundant ectoparasite (88.7%) in Bandar Abbas, Southern Iran, was Xenopsylla, found in *Rattus norvegicus* ( Kia *et al.*, 2009).

A previous study by Ristiyanto *et al.* had also found that female *Rattus tanezumi* had more ectoparasites than males, while more ectoparasites were in male Rattus exulans rats than in female ones ( Ristiyanto, Mulyono, Agustina, Yuliadi, & Muhidin, 2011). Riyanto also found that the most common type of flea found in house rats was *Xenopsylla cheopis.* This suggests the potential for disease caused by rats and their ectoparasites ( Riyanto, 2019). However, not all rats will have ectoparasites, as many aspects can influence their presence. One aspect that can affect the presence of ectoparasites in rats is the season. In the summer, due to dry and hot weather, rats and ectoparasites' presence is low ( Alahmed & Al-Dawood, 2001). The presence of rat ectoparasites that can potentially cause health problems leading to high mortality and morbidity must be controlled properly.

## Conclusion

Rats were mostly found in Cengkareng district, specifically in the East Cengkareng Sub-District, one of the districts with a high incidence of leptospirosis in West Jakarta. The most common rat species is *Rattus rattus.* The ectoparasite most commonly found in rats is the male flea *Xenopsylla cheopis.* Surveys, monitoring, and control of rats and ectoparasites are essential for the preparedness and development of an early warning system of possible diseases that they can cause. Future research should include data about the relative abundance index of trapping, a map of the trapped area, and information about the house/region.

## Data availability

### Underlying data

Dryad: Ectoparasites in rats and shrews data related to leptospirosis in West Jakarta. https://doi.org/10.5061/dryad.t4b8gtj18 ( Susanna, Nova & Rozek, 2021).

This project contains the following underlying data:

- Data 1. sav (dataset containing the region, name of species Rattus, gender, body length, tail length, rear length, feet length, earlobe length, head length, mamae, and weight.)
- Data 2. sav (dataset containing species of ectoparasite and gender)

Data are available under the terms of the Creative Commons Zero “No rights reserved” data waiver (CC0 1.0 Public domain dedication).
