# Supplementary material for: The existence and characteristics of rats and shrews in endemic leptospirosis areas and types of ectoparasites: a case study of West Jakarta, Indonesia
Source: F1000Res. 2021 Apr 30;10:335. [Version 1] doi: 10.12688/f1000research.47068.1 (PMC8649970; doi:10.12688/f1000research.47068.1)
Supplement: Peer review history

# Peer review: Reviewer response for version 1

**DOI:** 10.5256/f1000research.50145.r99700

**Published:** 2021-12-06

**Authors:** Rahayu Lubis

**Affiliations:** 1 Department of Epidemiology, Faculty of Public Health, Universitas Sumatera Utara, Medan, Indonesia

This research has complete data and methods that can be carried out elsewhere. This is very beneficial for the community and it is necessary to increase early awareness of vector-borne diseases by rats and ectoparasites so that public health prevention efforts can be carried out early on, because the rat population is always present in the human population, especially in densely populated environments.

 “The type of ectoparasite found in the rats was fleas. *Xenopsylla cheopis* was the most common type, at 83.3% and more fleas were male, at 66.7%. The most common rat species was *Rattus rattus*. The ectoparasite most commonly found in them was the female flea *Xenopsylla cheopis*.”

- This is an important finding because it is known that the types of ectoparasites found in rats are fleas, especially *Xenopsylla cheopis*, so early awareness of zoonotic diseases, especially bubonic plague, needs to be carried out in the community.

“The technique for catching rats involved using humane live traps, while the identification of the rats and ectoparasites was done in the laboratory.”

- This study follows ethical rules in animals.
- In the method of catching rats, it is stated that the traps are set in the afternoon between 15.00 and 17.00 WIB, and the rats are collected in the morning between 07.00 and 09.00. Within 14 hours, if the rat dies and is surrounded by ants because of the smell, will it still be examined as a research sample?

Is the work clearly and accurately presented and does it cite the current literature?

Yes

If applicable, is the statistical analysis and its interpretation appropriate?

Yes

Are all the source data underlying the results available to ensure full reproducibility?

Yes

Is the study design appropriate and is the work technically sound?

Yes

Are the conclusions drawn adequately supported by the results?

Yes

Are sufficient details of methods and analysis provided to allow replication by others?

Yes

Reviewer Expertise:

Epidemiology, infectious disease, HIV/AIDS

I confirm that I have read this submission and believe that I have an appropriate level of expertise to confirm that it is of an acceptable scientific standard.

# Peer review: Reviewer response for version 1

**DOI:** 10.5256/f1000research.50145.r91881

**Published:** 2021-09-06

**Authors:** Philip Samuel Paulraj

**Affiliations:** 1 ICMR-Vector Control Research Centre, Puducherry, India

This article is a good paper and approved for indexing. This publication has good epidemiological significance correlating the presence of ectoparasites found on rats with the prevalence of leptospirosis.

 A similar study of mine conducted in scrub typhus endemic areas in India showed higher numbers of ectoparasites belonging to mites, fleas, and ticks. Since this study showed very few ectoparasites only belonging to fleas, an extensive study should be planned to be undertaken in these areas to collect/record more ectoparasites. This study can be taken up as a pilot study for future in-depth studies (P. Philip Samuel, R. Govindarajan, R. Krishnamoorthi, and V. Rajamannar A study on ectoparasites with special reference to chigger mites on rodents/shrews in scrub typhus endemic areas of Kerala, India *Entomon*, 45: 4; 2020 ^1^).

 You could also mention this reference of mine under ectoparasites identification: “Philip Samuel P., Govindarajan R., Krishnamoorthi R., Venkatesh A. (2021). A rapid protocol for clearing, staining, and mounting of Arthropoda: Trombiculidae, Pediculidae, and Pulicidae. *North-western Journal of Zoology* *,*17(1):1-5 Article No. e201104” ^2^.

 After a scientific name is written in full in a publication, it is acceptable or customary to abbreviate the genus name by just using the first initial (or as recommended) and then full stop to represent the genus. These changes can be made in the entire manuscript.

 Please mention the rat identification method.

Is the work clearly and accurately presented and does it cite the current literature?

Yes

If applicable, is the statistical analysis and its interpretation appropriate?

Yes

Are all the source data underlying the results available to ensure full reproducibility?

Yes

Is the study design appropriate and is the work technically sound?

Yes

Are the conclusions drawn adequately supported by the results?

Yes

Are sufficient details of methods and analysis provided to allow replication by others?

Yes

Reviewer Expertise:

Medical Entomology

I confirm that I have read this submission and believe that I have an appropriate level of expertise to confirm that it is of an acceptable scientific standard.

## References

[1] : A study on ectoparasites with special reference to chigger mites on rodents/shrews in scrub typhus endemic areas of Kerala, India. *ENTOMON* .2020;45(4) : 10.33307/entomon.v45i4.572 285-294 10.33307/entomon.v45i4.572

[2] : A rapid protocol for clearing, staining, and mounting of Arthropoda: Trombiculidae, Pediculidae, and Pulicidae. *North-western Journal of Zoology* .2021;17(1) :1-5

# Peer review: Reviewer response for version 1

**DOI:** 10.5256/f1000research.50145.r84286

**Published:** 2021-06-11

**Authors:** Malina Binti Osman

**Affiliations:** 1 Department of Medical Microbiology and Parasitology, Faculty of Medicine, Universiti Putra Malaysia, Serdang, Malaysia

The study provided information on known reservoirs which are linked to leptospirosis. Even though the areas has been said to be endemic with leptospirosis, none of the epidemiological data is presented. It has been mentioned in the abstract that data on leptospirosis was retrieved from West Jakarta Health Office, but there is no data on leptospirosis in this study.

 In my opinion, some statistics work should be inserted to allow any determination on correlation between number of the reservoirs with the number of cases reported.

 I would suggest molecular work on determining the presence of the bacteria in the reservoirs should be done as well.

Is the work clearly and accurately presented and does it cite the current literature?

Yes

If applicable, is the statistical analysis and its interpretation appropriate?

No

Are all the source data underlying the results available to ensure full reproducibility?

Partly

Is the study design appropriate and is the work technically sound?

Partly

Are the conclusions drawn adequately supported by the results?

Yes

Are sufficient details of methods and analysis provided to allow replication by others?

Partly

Reviewer Expertise:

Epidemiology, biostatistics, tropical health

I confirm that I have read this submission and believe that I have an appropriate level of expertise to state that I do not consider it to be of an acceptable scientific standard, for reasons outlined above.
